# Influence of expiratory positive airway pressure on cardiac autonomic modulation at rest and in submaximal exercise in COPD patients

**DOI:** 10.1590/1414-431X20187180

**Published:** 2018-04-19

**Authors:** C. da L. Goulart, E.A. San Martin, K.M.K. Mansour, P.B. Schneiders, A.L.G. da Silva

**Affiliations:** 1 Iniciação Científica, Curso de Fisioterapia, Universidade de Santa Cruz do Sul, Santa Cruz do Sul, RS, Brasil; 2 Curso de Fisioterapia, Departamento de Educação Física e Saúde, Universidade de Santa Cruz do Sul, Santa Cruz do Sul, RS, Brasil; 3 Programa de Reabilitação Pulmonar, Hospital Santa Cruz, Santa Cruz do Sul, RS, Brasil

**Keywords:** Autonomic nervous system, HRV, COPD, Positive-pressure respiration, Exercise test

## Abstract

The aim of this study was to evaluate the effect of expiratory positive airway pressure (EPAP) on heart rate variability (HRV) indices at rest and during 6-min walk test (6MWT) in chronic obstructive pulmonary disease (COPD) patients. Fifteen moderate to severe COPD patients were randomized and evaluated with and without (Non-EPAP) a 5 cmH_2_O EPAP device. Respiratory rate (RR) was collected at rest (5 min), during the 6MWT (5 min), and at recovery (5 min). Indices of HRV were computed in the time domain, in the frequency domain, and nonlinear analysis. For EPAP and Non-EPAP during the 6MWT, we found an increased mean heart rate (HR) (P=0.001; P=0.001) while mean RR (P=0.001; P=0.015) and RR tri index decreased (P=0.006; P=0.028). Peripheral oxygen saturation (P=0.019) increased at rest only in the EPAP group. In EPAP, correlations were found between forced expiratory volume in 1 s (FEV_1_) and low frequency (LF) sympathetic tonus (P=0.05; r=-0.49), FEV_1_ and high frequency (HF) parasympathetic tonus at rest (P=0.05; r=0.49), lactate at rest and LF during the 6MWT (P=0.02; r=-0.57), and lactate at rest and HF during 6MWT (P=0.02; r=0.56). Through a linear regression model, we found that lactate at rest explained 27% of the alterations of LF during 6MWT. The use of 5 cmH_2_O EPAP improved autonomic cardiac modulation and its complexity at rest in COPD patients. Although it did not influence the performance of the 6MWT, the EPAP device caused alterations in resting lactate concentration with an effect on sympatho-vagal control during the test.

## Introduction

The control of the autonomic nervous system (ANS) of patients with chronic obstructive pulmonary disease (COPD) is influenced by the sympathetic and parasympathetic systems, peripheral oxygen saturation (SpO_2_), pulmonary stretch baroreceptors, and cardiac and pulmonary reflexes (1–3). In COPD patients the sympatho-vagal imbalance is caused by alterations in the ANS (4), as well as by the bronchoconstriction mechanism, hypoxia, hypercapnia, and systemic inflammation (3,5,6), resulting in a higher risk of morbimortality (7).

Noninvasive ventilation (NIV) methods are frequently used in this population with the primary goal of increasing ventilation and SpO_2_, decreasing respiratory complications and dyspnea symptoms, reducing respiratory muscle load and the intrathoracic pressure during rest and physical exercise, and incrementing their physical and functional performance. Scientific evidence suggests that the use of NIV can modulate autonomic control of heart rate (HR) (1,2,8).

Studies using bilevel positive pressure airway and continuous positive airway pressure (CPAP) have demonstrated that this method is effective for improving pulmonary ventilation, neural control of HR, and sympatho-vagal balance in COPD patients (2,9,10). Nicolini et al. (11) evaluated the effect of expiratory positive airway pressure (EPAP) on the 6-min walk test (6MWT) in COPD patients and found a beneficial effect of EPAP on their functional capacity. Müller et al. (12) highlighted that CPAP and EPAP reduce static hyperinflation in COPD patients, and that EPAP demonstrates equivalent effects to CPAP on static hyperinflation indirectly measured by inspiratory capacity and maximal inspiratory pressure (MIP). EPAP application relieves dynamic airway compression during expiration, reducing expiratory airflow limitation and thereby increasing inspiratory capacity in COPD patients (13,14).

No study has evaluated the effects of EPAP on the autonomic cardiac indices through the analyses of heart rate variability (HRV). It is known that linear and nonlinear HRV indices are widely accepted as important tools to determine cardiac autonomic changes (2–9). Linear indices of time domain such as mean respiratory rate (RR) interval (mean RR), RR interval standard deviation (STD RR), mean HR, and STD HR reflect HR fluctuations. Variables such as square root of the mean squared differences of successive RR intervals (RMSSD) and the HRV triangular index (RR tri index) reflect global HRV. In the frequency domain, low frequency (LF) and high frequency (HF) indexes indicate sympathetic and parasympathetic activity respectively, and LF/HF ratio indicates sympatho-vagal relation. The analysis of nonlinear components [approximate entropy (ApEn) and Shannon entropy] indicates ANS complexity (15). Therefore, our aim was to evaluate the effect of EPAP on the HRV indices at rest and during 6MWT in COPD patients. We hypothesize that a 5 cmH_2_O EPAP application would provide a positive effect on autonomic modulation in COPD patients both at rest as during submaximal exercise.

## Material and Methods

### Study design

We performed a cross-sectional and randomized cross-over study within the Programa de Reabilitação Pulmonar, Hospital Santa Cruz, Santa Cruz do Sul, RS, Brazil. All patients were formally invited to participate in the study by the responsible researcher and all volunteers signed an informed consent statement prior to participation. The study was approved by the Research Ethics Committee of the Universidade de Santa Cruz do Sul (protocol No. 1.514.705).

### Subjects

**Figure 1.**
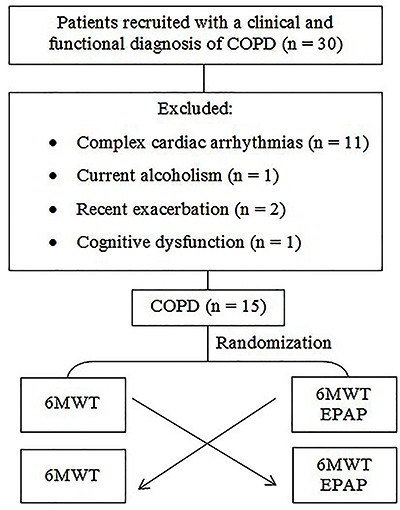
Flowchart of sampling and study phases. COPD: chronic obstructive pulmonary disease; 6MWT: 6-min walk test; EPAP: positive airways pressure.

Thirty patients with a clinical diagnosis of COPD by a pulmonary function test (forced expiratory volume in 1 s (FEV_1_)/forced vital capacity (FVC) ratio of 0.7; FEV_1_ 60% of predicted) with good cognitive function and without previous COPD exacerbation (15 days before the study) were included in the study. Patients with muscle-skeletal disorders or neurological conditions affecting the locomotor system in such a way that would preclude participation in the protocol, a clinical diagnosis of lung cancer, current alcoholism, complex cardiac arrhythmias, uncontrolled metabolic disease such as diabetes, thyroid changes, and systemic arterial hypertension (with or without the use of beta-blockers), or electrocardiogram alterations were excluded from the study (n=15; Figure 1).

### Measurements

Patients were evaluated in a laboratory with 22°C and relative humidity between 50 and 60%. They were instructed to avoid stimulants and alcoholic drinks and not to perform exhausting physical exercise the day before the test; they were also instructed not to smoke or use bronchodilators for 6 h before the test. Patients underwent a clinical evaluation to record gender, age, body mass index, smoking history, and COPD status by pulmonary function. The HRV signal was recorded at rest (5 min), during 6MWT (5 min) with and without EPAP, and during post-test recovery period (5 min). The order of the 6MWT with and without EPAP was determined by simple randomization (draw of an envelope) with a 30-min resting period between the tests (Figure 1).

### Pulmonary function

Pulmonary function was assessed using a digital spirometer (Microloop¯, MK8, Care Fusion, Germany), which provided measures of the FEV_1_. Spirometry was performed according to the recommendations of the American Thoracic Society (16) and the results were analyzed according to the values predicted by Pereira et al. (17). The classification of severity of airflow limitation in COPD was performed according to the Global Initiative for Chronic Obstructive Lung Disease (GOLD) recommendations and patients were classified as moderate (GOLD II), severe (GOLD III), or very severe (GOLD IV) (18).

### Respiratory muscle strength (RMS)

RMS was assessed using a digital manometer (MDI¯, MVD300, Brazil), which provided measures of the MIP and the maximum expiratory pressure (MEP). The assessment was performed according to the recommendations for the Brazilian population. The values were then compared with those described in the literature and reported as percentage of predicted values (19). The inspiratory muscle weakness was determined by an MIP <60 cmH_2_O (20).

### Submaximal exercise capacity - 6MWT

The 6MWT was carried out according to the guidelines of the ATS (21), in order to measure the distance walked during the 6-min test on a flat 30-m corridor. The subjects were instructed to walk as far as possible, at a constant speed. The 6MWT was developed based on the study by Holland et al. (22). The percentage of the predicted distance walked was calculated considering gender, age, height, and weight of each patient based on the equation of Enright (23). SpO_2_, HR, blood pressure, perceived exertion (using the Borg Scale), and distance walked (meters) were recorded at the beginning and end of every test as previously indicated.

### EPAP device

The EPAP device consists of a Spring Loaded¯ valve (Kit EPAP) connected to a 20-mm inner diameter and 100-mm tube and a mouthpiece (11). The positive expiratory pressure (PEP) set at 5 cmH_2_O, following previous findings on application of 5 cmH_2_O PEP, was safe and showed lower autonomic imbalance in COPD (2,11). Familiarization procedures were performed with the equipment and experimental protocols so patients could learn how to correctly perform the 6MWT with EPAP before the actual testing.

### Lactate

Blood lactate was obtained at rest, during 6MWT (test peak), and during post-test recovery period (third minute after the end of the test) with and without EPAP. The measurement of blood lactate concentration was made by collecting a drop of capillary blood on a validated portable lactometer (Accutrend Plus) (24).

### HRV

HR and RR intervals (iR-R) were recorded using a telemetric cardiac monitor (Polar¯ S810i, Finland). An elastic band (Polar T31 transmitter) was placed around the patient's thorax at the level of the lower third of the sternum, while the patient was in a sitting position; signals were continuously transmitted to the receiving unit by electromagnetic field. Recorded data were then transferred to Kubios HRV¯ analysis software (version 2.2, Finland) for subsequent analysis.

The signal processing was carried out as follows: at rest (5 min), during the 6MWT (we discarded the initial 60 s of data and selected the most stable signal, corresponding to the latter portion of the test), and post-exercise recovery (5 min) with and without EPAP. The HRV signal collected in the time domain provided mean RR, STD RR, mean HR, STD HR, RMSSD, and RR tri index, in the frequency domain were LF, HF, and LF/HF ratio, and nonlinear analysis provided the ApEn and Shannon entropy indices.

### Statistical analysis

Data were analyzed using the Sigmaplot¯ statistical package (version 11.0, Systat Software Inc., USA). Data were tested for normality through the Shapiro-Wilk test and presented descriptively as mean and SD (parametric) or as median and minimum and maximum interval (non-parametric). Analysis of variance (ANOVA) for multiple comparisons with Tukey's *post hoc* test was performed. Pearson correlation analysis was performed to investigate the correlations between variables. The analyses between the groups were performed by a Student's *t*-test. A linear regression model was used to determine the effect of the clinical variables on HRV parameters. Residuals were evaluated under the assumptions of normality, constant variance, and independence. P≤0.05 was considered significant.

## Results

Clinical characteristics of the patients included in the study are shown in Table 1. We observed a greater prevalence of patients with severe COPD in stage III and IV (60%), male gender, obesity, and only 33% with inspiratory muscle weakness.

**Table 1.** Clinical characteristics of chronic obstructive pulmonary disease (COPD) patients.

Variables	COPD (n=15)
Age (years)	62.6±7.8
Gender	
Male n (%)	12 (80.0)
BMI (kg/m^2^)	26.5±6.3
BMI classification, n (%)	
Underweight	4 (26.7)
Eutrophic	3 (20.0)
Obese	8 (53.3)
Pulmonary volumes	
FEV_1_ (L/s)	1.1 (0.7–1.6)
FEV_1_ (% predicted)	37.0 (25.0–68.0)
Staging (GOLD), n (%)	
Stage II,	6 (40.0)
Stage III	4 (26.7)
Stage IV	5 (33.3)
Respiratory muscle strength	
MIP (cmH_2_O)	73.0±28.8
MIP (% predicted)	72.6±28.6
MEP (cmH_2_O)	115.0±40.2
MEP (% predicted)	105.5±33.6
Inspiratory muscle weakness (MIP <60 cmH_2_O), n (%)	
Yes	5 (33.3)
No	10 (66.7)

Data are reported as means±SD and number (%): BMI: body mass index; FEV_1_: forced expiratory volume in 1 s; GOLD: Global Initiative for Chronic Lung Disease; MIP: maximum inspiratory pressure; MEP: maximum expiratory pressure.

The mean HR increased significantly during the 6MWT in both groups (P=0.001), while the mean RR (P=0.001; P=0.015) and RR tri index decreased (P=0.006; P=0.028) demonstrating an increase of the HR and reduction of global HRV compared to rest (Table 2). In the EPAP group, we found a significant result in Shannon entropy at rest (P=0.016), showing a better ANS complexity compared to Non-EPAP at rest.

**Table 2.** Comparisons within and between groups for all outcome measures.

Variables	EPAP Rest	Non-EPAP 6MWT	Recovery	Rest	6MWT	Recovery
SBP (mmHg)	116.6±11.1	137.3±21.2*	129.3±17.5	115.3±12.4	129.3±21.2	122.0±16.1
DBP (mmHg)	80.6±8.8	86.0±7.3	82.0±10.1	79.3±9.6	84.0±7.3	83.3±9.7
Lactate (mmol/L)	2.9±1.1	3.2±1.8	2.5±1.2	2.7±0.9	2.7±1.1	2.7±1.1
Borg - lower limb	7.3±2.0	9.5±3.1	8.1±2.3	7.3±1.7	9.0±3.0	7.4±2.6
Mean RR (ms)	729.3±123.8	556.1±109.1*	609.5±137.8	755.5±144.6	563.1±99.5*	708.4±130.0**
STD RR (ms)	11.2 (9.1–24.0)	6.8 (3.5–11.1)*	10.9 (7.5–17.0)	1.7 (1.3–2.4)	1.5 (1.0–2.1)	1.9 (1.4–2.2)
Mean HR (1/min)	84.4±13.4	111.9±22.3*	89.9±16.1**	82.1±15.3	109.8±20.0*	86.7±14.1**
STD HR (1/min)	1.7 (1.3–2.6)	1.4 (1.1–1.9)	1.8 (1.3–2.2)	1.7 (1.3–2.4)	1.5 (1.0–2.1)	1.9 (1.4–2.2)
RMSSD (ms)	16.6 (7.4–24.5)	6.3 (4.5–16.1)	12.5 (6.9–17.3)	18.2 (8.2–25.4)	8.6 (4.5–17.5)	10.3 (8.4–14.9)
RR tri index	4.3 (2.8–6.5)	2.3 (1.6–3.2)*	3.5 (2.7–5.2)	4.0 (3.2–5.6)	2.5 (1.7–3.2)*	3.8 (2.7–4.9)
LF (nu)	53.6 (35.5–82.1)	56.8 (33.8–70.1)	56.5 (48.9–75.7)	56.2 (31.8–76.2)	65.7 (40.9–75.3)	73.0 (51.2–83.8)
HF (nu)	43.6 (17.8–64.4)	43.1 (29.4–65.5)	43.4 (23.6–50.9)	43.4 (23.5–68.1)	32.9 (24.3–57.9)	26.6 (16.1–48.4)
LF/HF	1.2 (0.5–4.6)	1.3 (0.5–2.3)	1.3 (0.9–3.2)	1.2 (0.4–3.2)	2.0 (0.7–3.1)	2.7 (1.1–5.3)
ApEn	0.9±0.1	1.0±0	1.0±0	0.9±0	1.0±0.1	0.9±0.1
Shannon entropy	3.0±0.3	2.6±0.2	2.9±0.3	2.7±0.2^#^	2.8±0.3	2.8±0.2

SBP: systolic blood pressure; DBP: diastolic blood pressure; RR: respiratory rate interval; STD RR: RR interval standard deviation; HR: heart rate; STD HR: heart rate standard deviation; RMSS D: square root of the mean squared differences of successive RR intervals; RR tri index: HR V triangular index; LF: low frequency; HF: high frequency; nu: normalized units; LF/HF: ratio between LF power and HF power. ^*^P<0.05, rest *vs* 6MWT. ^**^P<0.05, 6MWT *vs* recovery. ^#^P<0.050 non-EPAP at rest *vs* EPAP at rest. Comparisons within groups were done with ANOVA and Tukey's *post hoc* analysis and between groups with Student's *t*-test.

**Figure 2.**
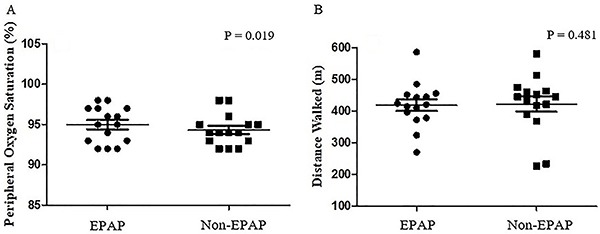
*A*, Peripheral oxygen saturation, and *B,* distance walked in the 6 min walk test in positive airways pressure (EPAP) and Non-EPAP groups of chronic obstructive pulmonary disease patients. Student's *t*-test was used for statistical analyses. Vertical lines indicate means and SDs.

EPAP showed an improvement of SpO_2_ at rest (P=0.019; Figure 2A). However, no significant difference was found in the distance walked in meters in 6MWT between groups (P=0.481; Figure 2B).

**Figure 3.**
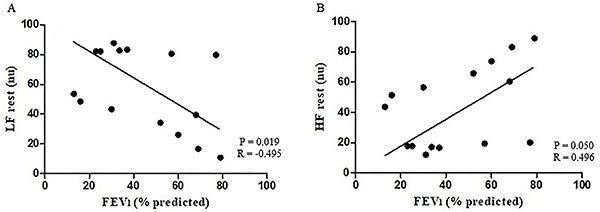
Relationship between lung function and heart rate variability index at rest. *A*, Negative correlation between LF (nu) and FEV_1_ (% of predicted). *B*, Positive correlation between HF (nu) and FEV_1_ (% of predicted). The Pearson correlation analysis was used for statistical analyses. FEV_1_: forced expiratory volume in 1 s; LF: low frequency; HF: high frequency; nu: normalized units.

Moderate correlations were found between airway obstruction and the HRV parameters at rest with EPAP (Figure 3), demonstrating the influence and importance of airflow obstruction over the sympathetic and parasympathetic modulation.

**Figure 4.**
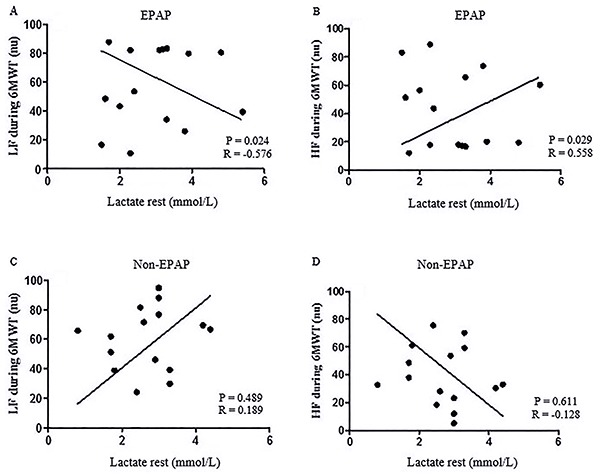
Relationship between lactate and heart rate variability index during 6-min walk test (6MWT). *A*, Negative correlation between LF (nu) and lactate at rest (mmol/L) with expiratory positive airway pressure (EPAP). *B*, Positive correlation between HF (nu) during 6MWT and lactate (mmol/L) at rest with EPAP. *C*, Positive correlation between LF (nu) during 6MWT and lactate (mmol/L) at rest in Non-EPAP. *D*, Negative correlation between HF (nu) during 6MWT and lactate (mmol/L) at rest in Non-EPAP. Pearson correlation analysis was performed. LF: low frequency; HF: high frequency; nu; normalized units.

An interesting finding in our study was the correlation between lactate at rest with HF and LF during 6MWT, demonstrating the direct influence of lactate at rest on sympathetic increase and parasympathetic reduction during 6MWT in COPD patients with EPAP (Figure 4A and B), that is, if resting COPD with EPAP show low lactate levels, during 6MWT they will have an expected ANS response with a predominance of sympathetic modulation. These findings were not found in Non-EPAP (Figure 4C and D).

These findings were confirmed through a linear regression model in which lactate at rest explained 27% of the alterations in LF during 6MWT (Table 3).

**Table 3.** Results of linear regression with expiratory positive airway pressure (EPAP) to determine the influence of lactate at rest on low frequency (LF; nu) during the 6-min walk test.

Variables	β coefficient	P
Constant	86.43	<0.001
Lactate at rest (mmol/L)	−12.06	0.027

Adjusted R^2^=0.273; F=6.25 (P=0.027). Equation to predict LF during 6MWT: 86.438 - (12,066* lactate at rest).

## Discussion

Based on our study, we highlight the following findings: a) EPAP had a beneficial effect on SpO_2_ and in ANS complexity, both during rest and compared to Non-EPAP; b) airway obstruction was associated with increased vagal modulation at rest with EPAP; c) resting blood lactate concentration was solely responsible for changes in sympathetic modulation during the 6MWT in COPD patients with EPAP. This finding can be attributed to higher SpO_2_ and better response of muscle metabolism.

The effects of EPAP on HRV complexity during rest in COPD patients are unprecedented and cannot be compared to other scientific studies. One of the main findings of our study with 5 cmH_2_O EPAP was the increase of SpO_2_, different from Müller et al. (12) study, in which they applied 10 cmH_2_O EPAP and found no improvement in SpO_2_. The main effect of the EPAP device consists in increasing expiratory flow and decreasing pulmonary hyperinflation (11). EPAP demonstrated equivalent effects to CPAP on static hyperinflation, improvements in inspiratory muscle strength, and inspiratory capacity (12). The device has been used in COPD patients to assist elimination of pulmonary secretions and induce a reduction in minute ventilation, respiratory rate, and physiological dead space (25,26). Reis et al. (2) highlights that treatment with a 5 cmH_2_O CPAP in COPD patients is safer and produces a better response in ventilatory parameters and autonomic balance than when compared with a 10 cmH_2_O pressure.

An interesting finding of our study was the association between VEF_1_ and parasympathetic and sympathetic modulation, as observed by HF and LF indices during rest with EPAP. Our findings contradict previous studies on different autonomic tests like postural changing, isometric contraction, and during dynamic physical exercise (4,7,27,28). These findings are consistent with the increased parasympathetic cardiac modulation intimately associated with airway obstruction from the bronchoconstriction of these patients (29). It is important to note that none of the studies used the EPAP device. We speculate that the beneficial effects of EPAP on bronchoconstriction reduction altered the autonomic cardiac modulation with lower parasympathetic modulation in patients with higher airway obstruction.

In our study, during the 6MWT with EPAP, the COPD patients presented an increase in SBP, increased HR and a reduction on global HRV, and we observed that these indices went back to normal values during recovery. A single study demonstrated that a reduction on HR occurred in moderate to severe COPD patients after the 6MWT with EPAP (11). The authors did not evaluate the autonomic cardiac modulation through HRV indices analysis, even though these indices are important markers of cardiac alterations (30).

Our study is the first to evaluate the association between blood lactate concentration at rest and sympathetic and vagal modulation during the 6MWT with EPAP, demonstrating that low levels of lactate at rest directly influence the autonomic sympathetic modulation during the 6MWT. This result was confirmed through linear regression. Lactate at rest and during low intensity exercise presents lower concentration, though it increases rapidly during exercise that requires an aerobic response (31), a result only found during 6MWT in the EPAP in our study. In COPD patients, the measurement of capillary blood lactate associated with the 6MWT can estimate muscular metabolic profile because its precocious increase before effort seems to be associated with muscular metabolic alterations, as loss of the oxidative activity of the skeletal muscles is observed (32).

It has been described in the scientific literature that during discontinuous resistance exercise in an elderly population, the integrative responses between muscular metabolism and autonomic cardiac control are more evident at aerobic metabolism to anaerobic transition during exercise (33), and these factors, as aerobic capacity, may have an influence on cardiac autonomic control (34,35). In coronary artery disease and chronic heart failure patients, HRV is a feasible tool in clinical practice to determine anaerobic threshold and it can be safe and appropriate to determine exercise intensity (36,37). Although having a different population and exercise protocol than our study, those findings may support the association between lactate at rest with autonomic modulation during 6MWT with EPAP in COPD patients.

The limitations that deserve to be listed are the rigid exclusion criteria and the difficulty in screening patients with 95% pure sinus beat. This study has a great significance and importance for the clinical management of patients with COPD, as the new results on EPAP application may be used in research and in the clinical field, as well as to assess the effects on interventions frequently used in pulmonary rehabilitation.

Finally, the use of 5 cmH_2_O EPAP improved autonomic cardiac modulation and ANS complexity during rest in COPD patients. Although it did not influence the performance of the 6MWT, EPAP caused alteration in resting lactate concentration with an effect on sympatho-vagal control during the test.
